# Utilization of Engineering Advances for Detailed Biomechanical Characterization of the Mitral–Ventricular Relationship to Optimize Repair Strategies: A Comprehensive Review

**DOI:** 10.3390/bioengineering10050601

**Published:** 2023-05-17

**Authors:** Antonia van Kampen, Jordan E. Morningstar, Guillaume Goudot, Neil Ingels, Jonathan F. Wenk, Yasufumi Nagata, Koushiar M. Yaghoubian, Russell A. Norris, Michael A. Borger, Serguei Melnitchouk, Robert A. Levine, Morten O. Jensen

**Affiliations:** 1 Division of Cardiac Surgery, Massachusetts General Hospital, Harvard Medical School, Boston, MA 02114, USA; 2 Leipzig Heart Centre, University Clinic of Cardiac Surgery, 02189 Leipzig, Germany; 3 Department of Regenerative Medicine and Cell Biology, University of South Carolina, Charleston, SC 29425, USA; 4 Cardiac Ultrasound Laboratory, Department of Cardiology, Massachusetts General Hospital, Harvard Medical School, Boston, MA 02114, USA; 5 Department of Biomedical Engineering, University of Arkansas, Fayetteville, AR 72701, USA; 6 Department of Mechanical Engineering, University of Kentucky, Lexington, KY 40508, USA; jonathan.wenk@uky.edu; 7 Department of Surgery, University of Arkansas for Medical Sciences, Little Rock, AR 72205, USA

**Keywords:** mitral valve, biomechanics, subvalvular apparatus, mechanical engineering, mitral valve simulation, force measurements, *ex vivo* modelling, computational modelling

## Abstract

The geometrical details and biomechanical relationships of the mitral valve–left ventricular apparatus are very complex and have posed as an area of research interest for decades. These characteristics play a major role in identifying and perfecting the optimal approaches to treat diseases of this system when the restoration of biomechanical and mechano-biological conditions becomes the main target. Over the years, engineering approaches have helped to revolutionize the field in this regard. Furthermore, advanced modelling modalities have contributed greatly to the development of novel devices and less invasive strategies. This article provides an overview and narrative of the evolution of mitral valve therapy with special focus on two diseases frequently encountered by cardiac surgeons and interventional cardiologists: ischemic and degenerative mitral regurgitation.

## 1. Introduction

**Figure 1:**
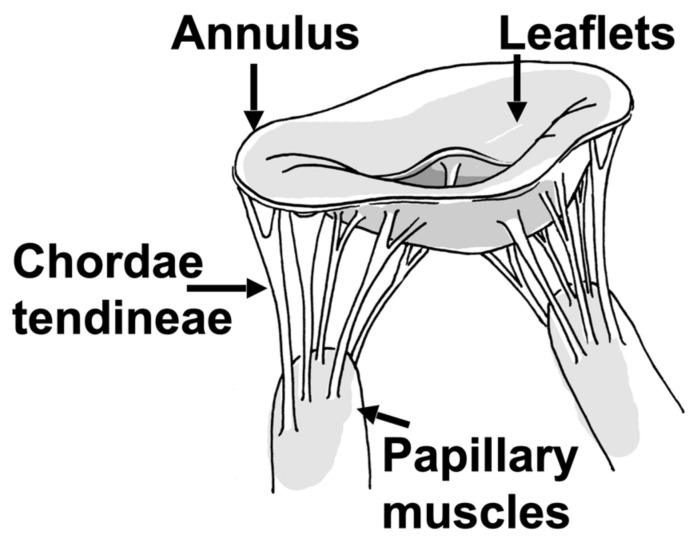
The mitral valve and the major structures and components.

The mitral valve (MV) and the left ventricle (LV) are interlinked through the sub-valvular apparatus, consisting of the two papillary muscles (PM) and the tree-like chordae tendineae spanning from each PM to both the posterior and anterior MV leaflets (Figure 1) [1]. This intricate system needs to interact in absolute harmony [2,3,4], as alterations within one of the integral components, caused by congenital, ischemic, degenerative, rheumatic, or infectious pathologies, can lead to damage and dysfunction of any of the other connected structures and, most importantly, their choreographed integrated function [1,5]. This becomes most relevant when an MV requires repair or replacement. Especially in the context of controversies about optimal therapeutic approaches, the exact biomechanical relationship of the MV and LV remains a topic of continuously growing interest. Recent engineering advances and the development of high-level ex vivo simulations have allowed for sophisticated investigations, and this review article aims to summarize the relevant discoveries made in this field, with special focus on ischemic and degenerative diseases.

## 2. Biomechanical Foundations of Mitral-Ventricular Interaction

### 2.1. Mitral Valve Modelling

Cardiac biomechanics has been a topic of scientific interest for decades and continues to draw attention. This is because engineering advances have provided enhanced imaging, measurement techniques, and predictive modelling capabilities. The available modalities can be divided into three categories: *in vivo*, *ex vivo*/*in vitro*, and *in silico*/computational modeling [6]. *In vivo* modelling remains the gold standard to create biomechanical readouts and correlate them with pathophysiologic changes that best depict human disease. However, techniques remain limited to those compatible with (temporary) survival of the research subject and can only be applied in the small number of disease models currently available. Moreover, direct visual assessment, real-time manipulation of intracardiac structures, and maintenance of controlled testing environments are challenging. Despite these limitations, several groups have successfully engineered instrumentation devices that can be used in preclinical *in vivo* models to examine the biomechanical MV–LV relationship, such as force transducers for radial annular, as well as chordal and papillary muscle forces [7,8,9,10,11,12].

**Figure 2:**
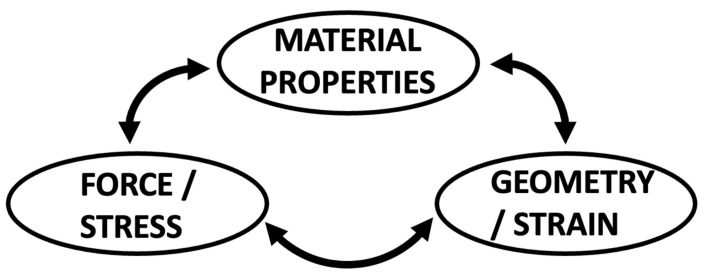
Geometry, force, and material properties are the three linked parameters that fully and completely describe the mechanics of any material, including biomechanics of biomaterials. If two of the three parameters are known, the third can be unambiguously determined. For example, the slope of the tangent at a point along the stress–strain relationship for a material is the stiffness property. This is called Young’s modulus for a linear elastic material. For all materials (linear and non-linear like MV tissue), this slope defines the unique relationship between length or length changes (geometry/strain), force or force per area (stress), and material properties.

Built on human and/or preclinically created *in vivo* data, computational modeling can extend tissue modifications beyond those observed or render *in vivo* models to test a broader range of properties, including the MV–LV interaction. Additionally, biomechanical interactions between the structural cardiac components can be created in a very controlled environment. However, computational modelling is inherently based on a series of assumptions regarding the mechanical properties and fatigue of different tissues and biological responses that researchers are not certain to be true with regard to human physiology [13,14]. Hence, the findings might not always be entirely translatable to *in vivo* conditions. Computational models also need to be informed and correlated with the biomechanical force balance of the different components of the MV in order to obtain the correct material properties [13,14,15,16]. This is based on the simple relationship between force, structure, and material properties. If two of these are known, the third can be unambiguously determined (Figure 2).

**Figure 3:**
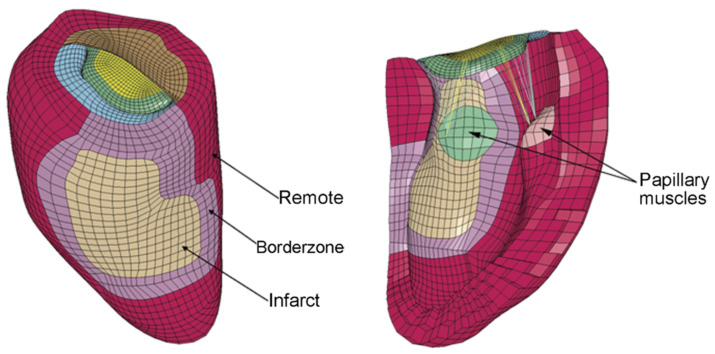
Finite element model of the left ventricle with mitral valve to provide insight into ischemic mitral regurgitation. Left: Exterior view of the left ventricle showing posterobasal myocardial infarction. Right: Interior view of the left ventricle with chordae connected to the papillary muscles. LV and MV built based on magnetic resonance tomography images of an ischemic MR ovine model using FindTags and FindContours software. Chordae tendineae reconstructed in approximation from anatomic images of an excised mitral valve. Surface meshes were applied to the model to replicate *in vivo* geometry. Border zone defined as steep decline in LV wall thickness between normal and infarcted zones. Tissue properties modeled as nearly incompressible, transversely isotropic, and hyperelastic [16].

Computational models have been created based on 2- and 3-dimensional echocardiography, as well as magnetic resonance imaging and computed tomography. These have generated biomechanical data on the healthy and diseased MV–LV relationship and different surgical techniques [17,18,19,20,21,22,23,24]. Furthermore, the models have predicted the observed role of MV elongation and papillary muscle anterior malposition in altering fluid interactions with the MV and driving LV outflow tract obstruction in hypertrophic cardiomyopathy [25,26]. Figure 3 shows the first published computational model of the MV–LV complex.

For *ex vivo* modelling, several groups have constructed and published inventive left heart simulators in which the MV can be investigated under approximated physiologic conditions [6,13,27,28,29,30,31,32,33]. In these models, MVs are explanted from porcine or ovine hearts and sutured onto annular and PM mounts that can be inserted into the *ex vivo* heart simulator. These simulators can be based on closed-loop pulsatile pump systems that can produce flow and pressure wave forms close to human physiologic conditions, where the pressure in the “LV” chamber mainly drives MV opening and closing [29,34,35,36,37,38,39]. More recently, systems focusing on static conditions where the geometry of the MV at peak systolic pressure is modeled have been developed [27,33]. While static PMs have presented a limitation of *ex vivo* MV modeling for decades, Imbrie-Moore et al. recently revolutionized the field by engineering robotic PM mounts for their *ex vivo* simulator, and were able to mimic 3-dimensional PM movement throughout the cardiac cycle [32]. Another highly innovative technique is used in a novel *ex vivo* platform published by Stephens et al.: the creation of a “left atrial” vacuum regulates MV closure and allows for manipulation of the “ventricular” side of the MV and subvalvular structures in an atmospheric pressure environment [27]. Within the published *ex vivo* simulators, hemodynamic conditions are usually controlled using flow and pressure sensors within the different components of the loop system. In addition, biomechanical data on the MV–LV system can be obtained using direct visual assessment, echocardiographic imaging, and dual-camera stereophotogrammetry with particle velocimetry, as well as strain-gauge force sensors implanted on the chordae tendineae or PMs [10,40,41,42,43,44].

It should be noted that the basic principles of fluid mechanics—applying conservation laws to control volumes within the circulation—have provided the Rosetta Stone for the noninvasive assessment of cardiovascular hemodynamics. Most notable are the simplified Bernoulli modification of the Navier–Stokes equation to derive pressure head loss across restrictive cardiac orifices by the conservation of energy [45,46,47], the quantification of regurgitant flows by the conservation of momentum [48,49], and the calculation of forward and regurgitant volume flows by the conservation of mass in laminar flows, including the vena contracta [50,51,52]. Applications of fluid mechanics also provided critical insight into evaluating regurgitation lesions based on jet size [53], highlighting the Coanda effect modifying the deceptively small size of the eccentric wall jets [54,55].

In the following sections, this review synopsizes characterizations of aspects of the MV–LV relationship that were obtained from all three of these modalities over generations of engineering innovations in this field [56,57].

### 2.2. The Physiologic Mitral–Ventricular Relationship

The basic anatomy of the human MV–LV apparatus has long been known in humans, while the functional anatomy, dynamics, and biomechanics have only been characterized comprehensively more recently, leveraging engineering biomechanical measurements and computational simulation techniques.

#### 2.2.1. Mitral Valve Leaflets

**Figure 4:**
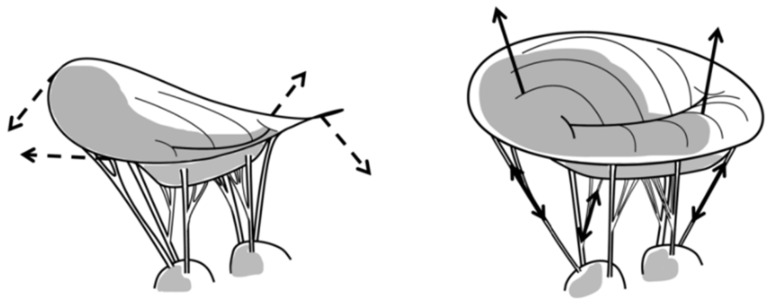
Schematic representation of the biomechanical effects of mitral valve flattening and forces. Left, normal mitral annular saddle shape; dashed arrows indicate forces inducing annular flattening. Right, annular flattening with mitral leaflet prolapse; arrows indicate increased out-of-plane forces transmitted to chordae and certain parts of the leaflets [4].

Using radiopaque markers and video fluoroscopy, Krishnamurthy et al. were able to show vast differences between explanted *in vitro* and *in vivo* MV leaflet properties, with significantly stiffer leaflet tissue *in vivo*. They furthermore detected significant *in vivo* anisotropy of the leaflet tissue, with a higher stiffness circumferentially than radially [58]. This was confirmed for both leaflets in stereophotogrammetry-derived 3-dimensional strain analyses of the anterior and posterior MV leaflets *ex vivo*, in which 5 × 8 marker arrays were placed on the leaflet centers. A major principal strain was found to be directed radially, while a minor principal strain was oriented in the circumferential direction. Overall, strain of both anterior and posterior leaflet showed a rapid increase during systole, followed by a plateau for approximately 200 ms (at 70 bpm) and then a decrease of strain when the valve aperture is open [40,41]. Figure 4 illustrates the biomechanical relationship between the leaflets, annulus, and subvalvular structures.

MV disease often results in increased chordal tension in a flattened annulus with prolapsing leaflets [4]. If the transmitral pressure across the membrane is constant and the radius of curvature is decreased in the prolapsing valve leaflet, then the tension in the leaflet tissue is paradoxically smaller in certain areas of the valve, according to Laplace’s law [4].

**Figure 5:**
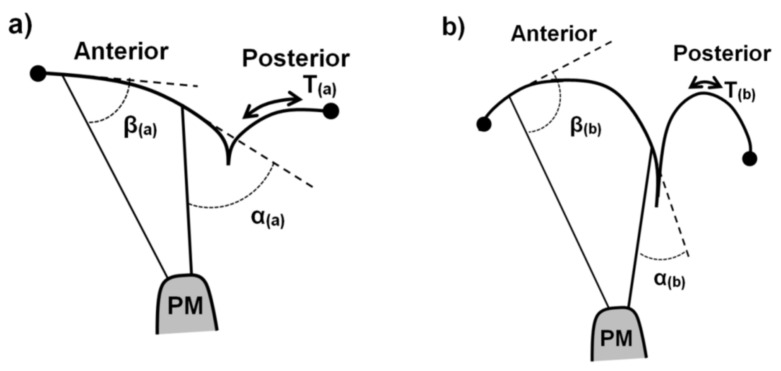
Simplified schematic illustration of (**a**) healthy vs. (**b**) prolapsing billowing mitral valve leaflet coaptation. Only two chords inserted into the anterior leaflet (basal and secondary positions) are shown for clarification. α and β illustrate angles between chordal insertion and leaflet tangents, respectively. T nominates tissue tension, which is smaller when the radius of curvature is smaller. This paradox leads to an unhealthy force balance in the valve.

The issues with prolapsing leaflets arise when the natural balance of chordal forces to seat the valve is disturbed. If the total annulus area remains constant and certain areas of the valve experience less strain and tension, it is expected that other areas of the valve must experience a higher degree of tension in the tissue structures (Figure 5). As will be shown below in Section 2.2.3, the leaflet shape is highly impacted by the chords. Considering the collagen direction across the surface of the leaflets [59,60], they are perpendicular to the LV/leaflet convex shape in the septal-lateral direction, but parallel in the orthogonal direction, where the LV/leaflet concave shape dictates a higher load. These shapes are also visible in 2D and 3D echoes across species (human, porcine, ovine.) Mitral valve prolapse is further described in Section 4.1.

#### 2.2.2. Mitral Valve Annulus

The non-planar saddle shape of the mitral annulus during systole was first discovered by Levine et al. [61]. Using 3-dimensional reconstructions of echocardiographic images, Flachskampf et al. then defined normal mitral annular dynamics throughout the cardiac cycle based on least-square planes and centroids using custom software. Healthy mitral annuli showed a 23.8% change in a cross-sectional area between end-diastole and end-systole, and systolic atrially directed movement of anteroseptal and posterolateral annular segments, while septal and anterolateral segments moved in the ventricular direction, creating the shape of a saddle during systole [62]. The Yoganathan and Gorman groups then suggested through computational and *ex vivo* modelling that this nonplanarity, as opposed to a more flattened annulus, can simulate reduced leaflet stress and forces acting out-of-plane on the chordae tendineae during systole [40,41,63,64]. In addition to annular dynamics, the force direction and distribution of the annulus has been examined. Using novel force transducers able to measure radial in-plane and out-of-plane forces in healthy large animal models, the Jensen group has demonstrated cyclic differences of septal-lateral and transverse annular forces from LV diastole to mid-systole, paralleling the increase of LV pressure during isovolumetric contraction [11,65]. The out-of-plane force balance favors the saddle-shaped annulus [66], with rigid annuloplasty rings absorbing forces in both the septal-lateral and commissure–commissure direction [66] and saddle-shaped rings, also improving coaptation [67].

#### 2.2.3. Chordae Tendineae

**Figure 6:**
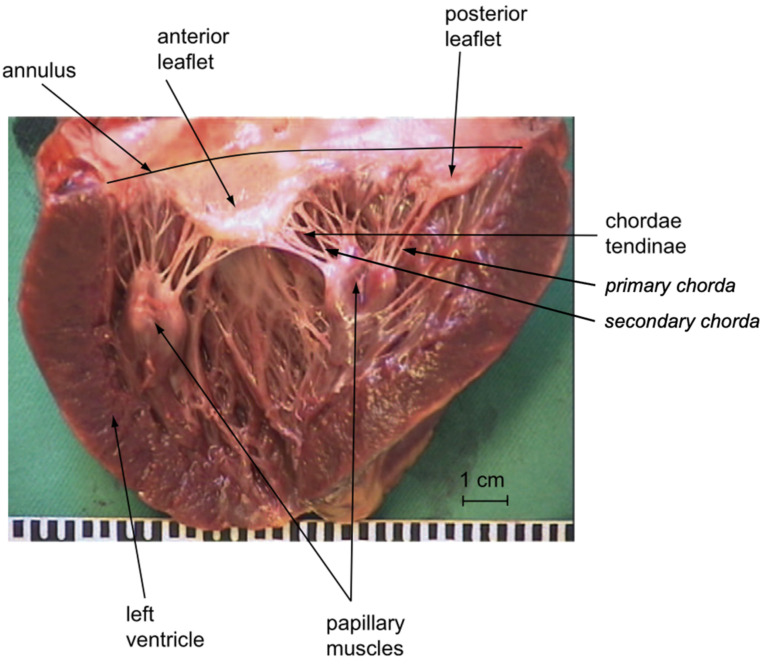
Chordal distribution and insertion pattern of the mitral valve viewed from the ventricular side in a human heart [70]. The chordae insert around roughly two-thirds of the annulus near the base of the leaflets. The anterior and posterior mitral valve leaflets are shown here, and the subvalvular apparatus exposed through an anterior ventricular transsection. The multi-headed organization of the PMs from which the chordae originate at the fibrous membranes can be appreciated. Reproduced with permission.

Chordae tendineae arise from the fibrous membrane of the base or from multiple heads of both PMs, and fan out as tree-like structures before inserting into both MV leaflets at different regions, relating to their role in the PM–MV interaction. Strut chordae insert in the central leaflet region, marginal chords at the free edge, and basal chordae at the annular edge (Figure 6) [68,69].

**Figure 7:**
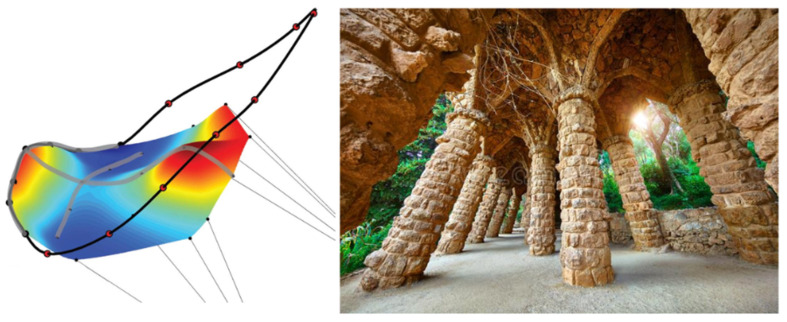
The mitral valve anterior leaflet (**left**) is self-supporting with the convex shape towards the left ventricle in the septal-lateral direction, similar to Gaudi’s Park Güell in Barcelona, where the structures are self-supporting under the weight of gravity (**right**) [74]. The collagen direction in the anterior leaflet is parallel to the circumferential direction, which is concave to the ventricular pressure, and a significant part of these fibers terminate in the strut chordae to help set the shape of the leaflet in early systole.

Marginal chordae are frequently called primary, and strut and basal chordae are summarized as secondary chords (Figure 6). MV chordae are a key element of the MV–LV complex, and their integrity is not only crucial for optimal MV function, but also for the maintenance of optimal geometry and global function of the LV [2,8,71]. As leaflets and chords model from the embryonic state as the stretching of cushions in the developing heart, Lam et al. has described “cleft chordae” that split shortly after arising from the PMs into different zones of the leaflets, and produce an interesting potential effect on MV closing [72,73]. The chordal network has an important function to set the leaflets into a specific initial 3D geometric configuration (stiff hyperbolic paraboloid anterior leaflet saddle shape, radially convex, circumferentially concave to the LV) at the time of the initial systolic increase of LVP [74]. This enables the leaflets to undertake a near self-supporting structure under the high systolic pressure of the ventricle. Similarly, the amazing arches in Gaudi’s Park Güell in Barcelona are self-supporting under the weight of gravity acting as the equivalent force of pressure (Figure 7). Any disturbance of this shape, including prolapse, impacts the self-supporting nature of the valve leaflets, and increases the load on the anchor points, including the subvalvular apparatus.

Further supporting this concept, it has been known for almost half a century that the chordae straighten under high strain rates during the closing phase of the cardiac cycle to maintain the leaflet shape and coaptation [75]. To investigate the mechanical responses of MV chordae during the cardiac cycle, Ritchie et al. mounted porcine MVs in an *ex vivo* left heart simulator and utilized marker tracking of the strut chordae. They found an average chordal strain rate of 75.3% during systolic closure and −54.8% during diastolic opening, with a constant plateau during valve closure [76]. As a first attempt to quantify chordal forces *in vivo*, Nielsen et al. engineered small, C-shaped strain gauge force transducers implantable into a transected MV chordae with standard suturing techniques [10]. Applied *in vivo*, they found that secondary chordae experience higher tension forces than primary chordae at different LV pressure conditions, with 0.7 and 0.2 N at a mean arterial pressure of 100 mHg, respectively [43]. These results were confirmed *ex vivo* by Paulsen et al., who developed novel fiber Bragg grating force sensors implantable on native and artificial chordae tendineae, which were able to detect mechanical strain with a resolution of 0.1 microstrain. The absolute values they measured at 100 mHg mean arterial pressure were 0.12–0.17 N for primary and 0.6–0.74 N for secondary chordae, with higher values for those to the anterior than those to the posterior leaflet and correlation of LV pressure and chordal forces. Moreover, using porcine MVs in the Stanford *ex vivo* simulator, they detected the force profiles of the primary and secondary chordae with a rapid primary peak at valve closure, rather stable forces while the MV remains closed, and a rapid decrease upon valve opening [42]. He et al. utilized an alternative method of chordal force measurement by implanting commercially available AIFP4 transducers (MicroStrain Inc., Williston, VT, USA) into incisions of single chordae tendineae and found comparable tension forces for strut and marginal forces as the other groups, as well as a correlation of the LV pressure and chordal forces [77]. Finally, Sedransk et al. conducted tensile testing of porcine MV chordae and detected that primary chordae ruptured at 68% less load and 28% less strain than secondary chordae, and posterior leaflet chordae ruptured at 43% less load and 22% less strain than anterior leaflet chordae [78].

#### 2.2.4. Papillary Muscles and Adjacent Myocardium

**Figure 8:**
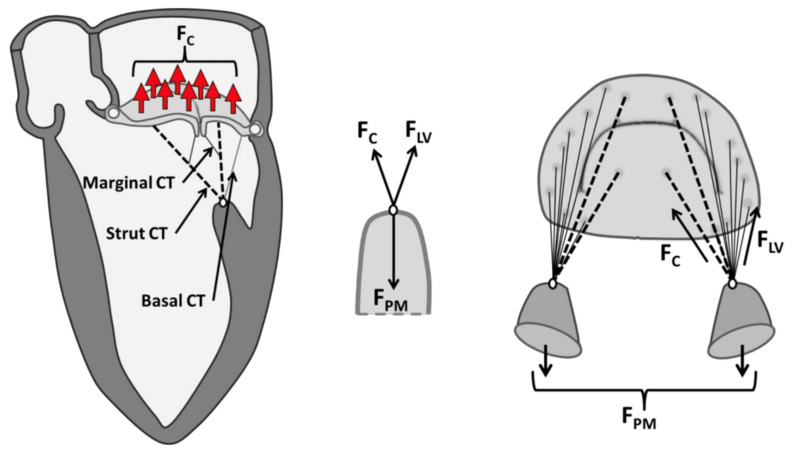
Separating the components of the PM force into the closing force (F_C_) and a potential valvular–ventricular interaction force (F_LV_). Reprinted with permission from [8]. 2013, Elsevier.

The anterolateral and posteromedial PMs both give rise to chordae tendineae inserting into both MV leaflets. The “multi-headed” nature of the PMs is potentially critical to ultimately understanding the mechanics of the system in order to support all the geometric requirements for the valve. It has been shown that PMs are parallel to the LV major axis, and the distance between the MV annulus and PM tips remains constant throughout the cardiac cycle due PM contraction and shortening during systole, while the inter-PM distance is significantly shorter at end-systole compared to end-diastole, as shown by experimental data leveraging engineering techniques [79,80,81]. Imbrie-Moore et al. analyzed 3-dimensional PM dynamics in detail using computed tomography images of healthy patients and were able to translate those into six-axis papillary muscle robots that can be utilized in *ex vivo* simulation [32]. Askov et al. were the first to conduct a comprehensive analysis of the total force transfer between MV and LV via the two PMs. They developed dedicated strain gauge force transducers [82] and implanted them into the PM tips of healthy pigs. They postulated that a force equilibrium consists of apically directed PM contraction force and MV closure force + MV–LV interaction force, with *MV closure force = mitral annular orifice area x transmitral pressure* [8,29] and *MV–LV interaction force = PM force − MV closure force* (Figure 8).

They found that the calculated MV closure and directly measured PM forces rapidly increased during early systole, peaking shortly before mid-systole, plateauing in mid- and late systole, and then rapidly decreasing at end-systole/early diastole. The normalized peak PM forces were 5.9 and 5.8 N for the anterior and posterior PMs, respectively, at baseline, with close correlation to the transmitral pressure [8]. These force measurements are in line with recent *ex vivo* work by Park et al., which shows a maximum force of 6 N at 100 mm Hg mean arterial pressure averaged over both PMs [44]. In addition, it is worth noting from Askov’s work that the valve is closed even though the pap forces are near zero. This again suggests that the chordae are primarily setting the geometry of the leaflets. In addition, the rising and falling forces during systole suggest that this force is acting primarily on the annular attachments of the chordae, contributing to the saddle shape in the valvular–ventricular interaction, with the leaflets not being pulled apart by these forces in the closed valve [74].

## 3. Chronic Ischemic Mitral Regurgitation

### 3.1. The Ventricle Tethers the Valve

**Figure 9:**
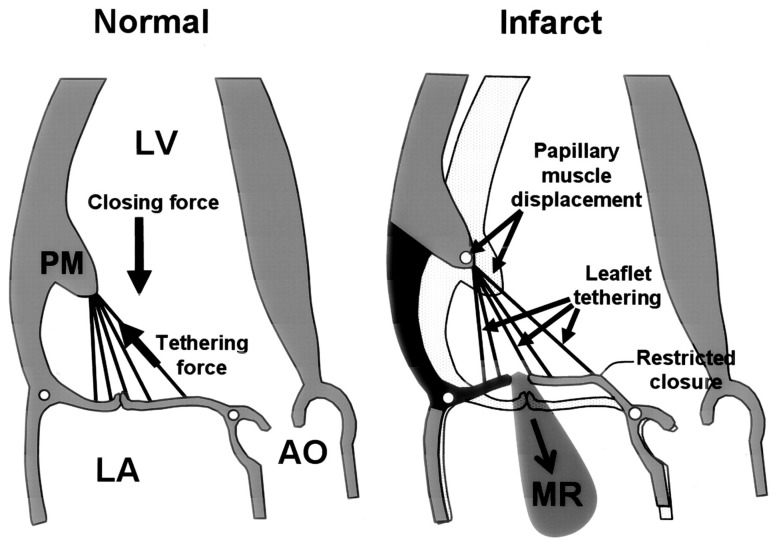
Mechanism of chronic ischemic mitral regurgitation due to displacement of the infarcted inferior LV wall and the posteromedial papillary muscle. Reprinted with permission from [83]. Copyright 2000, Wolters Kluwer/American Heart Association.

Changes in LV dimensions or geometry associated with persistent damage after myocardial infarction (MI) of the inferior LV segments lead to displacement of the papillary muscles (PMs) and subsequent tethering of the MV leaflets [1]. This condition is exacerbated by annular dilatation that occurs as part of the LV dimensional changes [35]. As a consequence, leaflet coaptation is incomplete, which causes ischemic MV regurgitation (IMR, see Figure 9).

Using an advanced *ex vivo* simulator and specifically engineered sigmoid-shaped rods, He and colleagues were the first to show that artificial displacement of the PMs by just 10 mm in five different directions (apical, lateral, posterolateral, lateral + apical, and posterolateral + apical displacement) produces tenting of the MV leaflets and regurgitation, with incremental severity for increased PM displacement distances in the bidirectional displacement, and less severity for isolated apical displacement [34]. Importantly, this model reproduced the biphasic early and late systolic peaks of the MR orifice in ischemic hearts [84]. Three-dimensional PM displacement was further investigated *in vivo* by Bothe et al. and Tibayan et al., each using dedicated radiopaque markers placed on the PM tips and the mitral annulus before induction of a posterolateral MI. In their ovine and porcine models, they detected posterolateral displacement for the posteromedial papillary muscle, but no apical displacement [85,86]. Kalra et al. conducted a comprehensive analysis of papillary muscle dynamics as an indicator of intact vs. impaired MV–LV interaction using cardiac magnetic resonance imaging [81]. Comparing patients with mild vs. moderate/severe IMR with similar inter-PM distances at end-diastole, they found that in mild-IMR patients, the inter-PM distance was shortened by 11.5 mm on average, leading to localization of both PMs within the mitral annular projection at end-systole. In patients with moderate/severe IMR, the inter-PM distance was shortened significantly less (9.6 mm on average), placing one or both PMs outside the annular projection, causing leaflet tethering and malcoaptation. In addition, they created porcine models with sub-PM vs. non-PM MI and found that sub-PM MI decreases inter-PM shortening and leads to more IMR compared to non-PM MI [81].

### 3.2. Annular Mechanics and Remodeling

Ischemic mitral regurgitation (IMR) is increased by added annular dilation, a common feature of post-MI LV remodeling, which has been modeled in different ways. As early as 1996, Kunzelman et al. conducted finite element modeling of the MV and found that increasing the annular circumference by 18% led to significantly increased leaflet stress and delayed and incomplete coaptation [87]. Using an *ex vivo* left heart simulator with a D-shaped adjustable mitral annulus that could be expanded from 5.5 cm^2^ to 13 cm^2^, He et al. showed that in the absence of PM displacement, annular dilation causes significant increases in the regurgitant volume and effective regurgitant orifice area at an annular dilation of >1.75× its baseline area, while in combination with especially asymmetric PM displacement with tethered leaflets, only a 1.5× increase in the annular area was needed to cause these significant changes. Compensation of annular dilation was therefore associated with mobile/untethered leaflet reserve (coapting leaflet portions) [35], which is further exacerbated by leaflet fibrosis and insufficient adaptational leaflet growth, as shown by a series of experimental 3-dimensional imaging studies [88,89,90]. Bioengineering studies have also confirmed the fundamental cellular changes in phasically stretched MV cells, modeling the tethered valves that are pro-fibrotic and induce stiffening that further impairs coaptation [60,91,92,93]. Noninvasive measurement of the leaflet strain is now possible to explore its role in the MV–LV coaptation dynamic [94,95]. Several groups are actively exploring how these mechanical leaflet changes compound tethering to augment MR and its associated heart failure and mortality [96,97,98,99,100].

**Figure 10:**
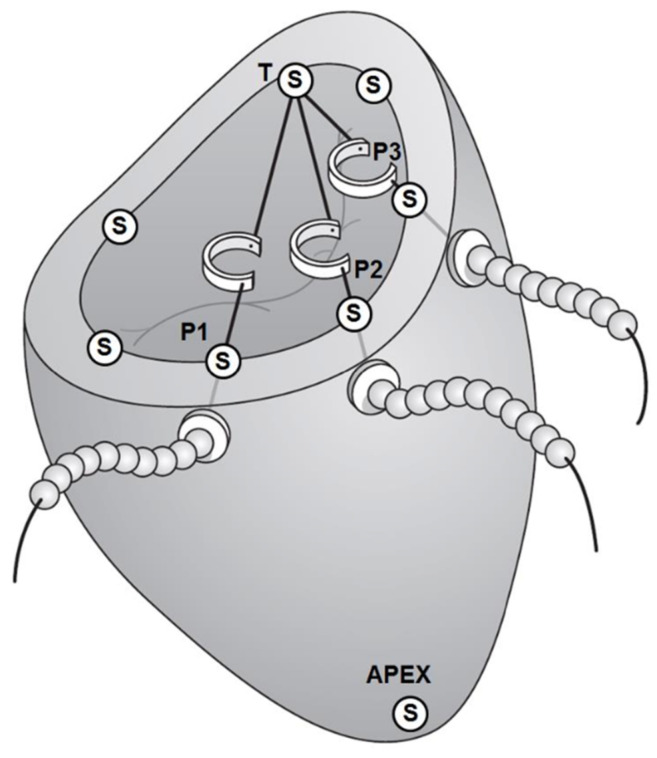
Measuring MV annulus downsizing forces. The sutures are anchored at the posterior trigone (T) and externalized at the posterior scallops (P1, P2, P3). Seven sonomicrometry crystals (S) are implanted at the annulus and one at the apex (S) of the left ventricle. Reprinted with permission from [105]. Copyright 2014, Elsevier.

To examine the annular dilation in IMR in more detail, Rausch et al. used eight markers implanted around the mitral annulus of sheep with inferior myocardial infarctions and obtained four-dimensional marker coordinates of three consecutive cardiac cycles by semi-automated radiographic image digitization and processing. They calculated the Bernstein coefficients to determine the marker position in relation to mathematically constructed spline curves for any time point during the cardiac cycles [101]. The strain/stretch was then calculated using local tangent vectors of marker displacement, and the overall relative curvature as the difference between the absolute curvatures of the IMR and the baseline annulus. They detected 15.2% and 14.2% increases in the septal-lateral and inter-commissural distances, respectively, and 10.4% and 18.4% increases in the septal and lateral perimeters, respectively. The annular area increased by 35.4% and there was a slight decrease in the annular saddle height. Using a strain analysis, they detected that all annuli were stretched in chronic IMR vs. a baseline condition, with a 5% strain in the septal area, up to 50% strain in the lateral segments, and an average location of the peak strain in the lateral-posterior segment, corresponding to the location of the myocardial infarction [101]. Tibayan et al. and Bothe et al. confirmed the significant increase in septal-lateral annular diameter in porcine and ovine IMR models using radiopaque markers placed around the annulus [85,86]. To investigate the changes in the mitral annular forces *in vivo*, Skov et al. applied a dedicated force transducer able to measure simultaneous in-plane and out-of-plane forces in an established porcine model [11]. Downsizing the MV annulus with an annuloplasty procedure is a popular surgical procedure to increase leaflet coaptation and restore valvular competency in functional disease. A wide selection of devices exists in many different sizes, shapes, and flexibilities. The optimal device for the downsizing procedure is often debated. The approaches that have been suggested to manipulate the MV annulus in innovative ways include percutaneous transvenous mitral annuloplasty, percutaneous septal-lateral diameter shortening, and ventricular approaches [102,103,104]. For example, in an acute porcine study [105], three traction sutures were anchored at the posterior trigone. Each suture suspended a miniature force transducer over the annulus and was externalized at the P1, P2, and P3 scallops, respectively [105]. The sutures were externalized and instrumented with a custom device capable of downsizing the mitral annulus in known increments (Figure 10).

Using these previous data, Imbrie-Moore et al. constructed a spring dilation system with individual independent anchors mounted along the annulus, using two different types of springs for the septal-lateral and the transverse directions. With a 30% increase in the annular “septal-lateral” distance and PM displacement by 10 mm apically and 10 mm laterally, they created an *ex vivo* model of IMR with a regurgitant fraction of 35% on average, which was subsequently used to examine different repair techniques [106]. Overall, advances in engineering and the incorporation of prior evidence into contemporary *ex vivo* and *in vivo* biomechanical analysis techniques have led to increasingly more granular and realistic models of IMR that can serve to validate therapeutic options.

### 3.3. Implications for Existing and Future Surgical and Interventional Approaches

The evolution of the IMR therapy is a stellar example of how engineering-guided experimental data have informed clinical practice and led to drastic changes in patient management. The former gold standard of undersized surgical mitral annuloplasty (SMA, either at the time of coronary revascularization or as a singular operation) for IMR has been questioned for years and has essentially been deemed insufficient based on clinical and experimental evidence. Engineering-based simulations of IMR repair have contributed greatly to uncover the reasons for the clinically reported unacceptably high failure rates of isolated SMA in IMR [107]. Wong et al. conducted modeling of SMA in a finite-element model of IMR based on cardiac MRI in an established ovine disease model. Finite-element 3-dimensional meshes of two different annuloplasty rings were created via digitized and processed photography. They showed that undersized SMA eliminated IMR and significantly relieved anterior and posterior leaflet stress and LV basal myofiber stress, as well as chordae tendineae stress [108]. This was confirmed by Siefert et al. using the Georgia Tech *ex vivo* simulator: they showed that with progressively more undersized SMA, the IMR and chordal forces were reduced and the coaptation length increased [109]. While these studies indicated sufficient short-term effects of undersized SMA, a multitude of experimental and clinical imaging studies exposed the shortcomings of this technique *in vivo*: in an ovine study using dedicated radiopaque markers and computational 3-dimensional reconstructions, posterior leaflet restriction was exacerbated after an undersized SMA was performed [110]. Furthermore, due to the progressive nature of LV remodeling causing IMR, SMA quickly becomes insufficient when the PMs are further displaced, and the leaflets are progressively tethered. PM displacement from outside the SMA projection was revealed as a prognostic factor of residual and recurrent IMR [1,111,112,113,114,115]. Imbrie-Moore et al. elegantly demonstrated this in the Stanford *ex vivo* simulator using an IMR model consisting of PM dislocation and annular dilation. Initially, the elimination of IMR by undersized SMA was reversed by further PM dislocation outward and apically [116].

**Figure 11:**
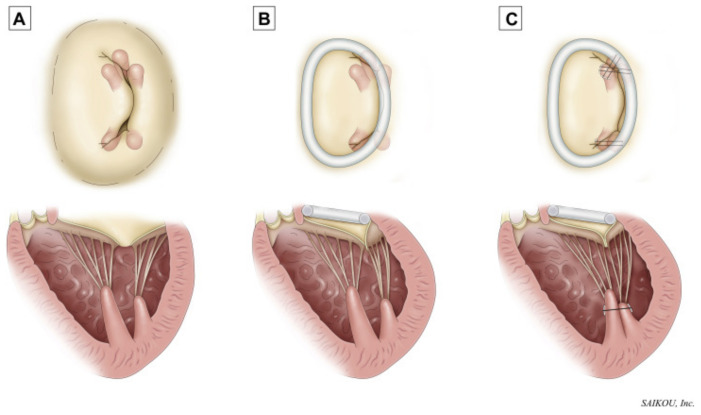
Surgical approach to (**A**) ischemic mitral regurgitation with (**B**) annuloplasty alone or (**C**) annuloplasty plus papillary muscle approximation. Reprinted with permission from [130]. Copyright 2020, Elsevier.

To improve surgical outcomes, adjunctive durable restoration of a physiologic inter-PM distance by surgical approximation after an undersized SMA was performed was proposed and tested extensively *ex vivo* with promising results by several groups: the coaptation length was increased, and the chordal forces and tenting area were reduced [106,116,117,118]. To achieve PM re-alignment without any intracardiac surgery, several groups have engineered innovative devices that could restore LV geometry and the inter-PM distance by external compression of the inferior LV wall. They showed that PM re-approximation using balloon patches, myocardial wrapping in mesh material, or highly fine-tuned external cardiac support devices without SMA successfully restored MV coaptation and even contributed to reverse LV remodeling, thus, long-term prevention of recurrent IMR after inferior MI (see Figure 11) [111,112,117,118,119,120,121,122,123,124]. Some devices have made it to first-in-man application and the technologies are expected to further evolve and become available in a more wide-spread fashion. Due to above-mentioned shortcomings of SMA and the still experimental nature of sub-valvular repair techniques, MV replacement has been considered a more durable option to treat IMR in recent years; however, as engineering-based approaches to reduce tethering advance, we can reasonably anticipate progress toward a more physiologic repair and realignment of the native mitral valve. Based on the above-explained importance of the chordal apparatus for LV (and not only MV) function defined by advanced analyses, partial preservation of the chordal apparatus during MV replacement is recommended univocally in the current guidelines [125,126]. Furthermore, our more thorough understanding of the MV–LV interaction in chronic IMR pre- and post-repair has led to development and *in vivo* testing of novel repair approaches, such as the severing of secondary chords to relieve PM tethering, reduce leaflet tension, and improve leaflet mobility and coaptation [127,128,129].

Finally, the emergence of transcatheter devices for MV repair, especially the MitraClip device used for transcatheter edge-to-edge repair (TEER) and its disputable success in IMR, has re-attracted attention towards geometrical and biomechanical effects of such new approaches [131]. In a finite element study based on ovine IMR models, Zhang et al. investigated the mechanical effects of a virtual MitraClip that was constructed based on the MitraClip NTR (Abbott Vascular, Santa Clara, CA) and found it to deform the annulus by shortening the anterior–posterior diameter, leading to a more elliptical shape, which increased end-systolic and end-diastolic leaflet stress and tissue strain in the sub-annular myocardium [132]. Significant and unphysiological increases in the leaflet stress and annular deformation after MitraClip implantation were confirmed by Kong et al. and Caballero and colleagues in finite element and fluid-structure interaction models of IMR patients [133,134]. In a follow-up study, Caballero et al. directly compared the MitraClip with the PASCAL device (Edwards Lifesciences, Irvine, CA) and found PASCAL to cause less annular deformation and decreases in leaflet stress and strain, while the MitraClip increased these factors in accordance with the previous studies [135]. These increased myocardial stresses induced by restrictive annuloplasty and clip techniques may have the capacity to drive the observed post-intervention LV remodeling and dilation that underlies recurrent IMR [115,132,136]. Based on the odyssey of IMR repair techniques to date, it is clear that biomechanical analyses will continue to play a crucial role in optimizing the current approaches and developing new strategies tailored to the unique demands of an optimal MV–LV interaction in patients with IMR.

## 4. Mitral Valve Prolapse

### 4.1. The Valve Pulls on the Ventricle

Mitral valve prolapse (MVP) is categorized by enlargement and mechanical incompetence of one or both of the mitral valve leaflets, leading to superior displacement of the leaflets during ventricular systole, and often failure of leaflet coaptation, causing mitral regurgitation [137]. Though commonly thought of as a condition that develops and worsens during adulthood, MVP occurs within many genetic syndromes, which suggests that there is a developmental origin to this disease [138]. Furthermore, genetic discoveries stemming from family studies of non-syndromic MVP and investigated using murine model systems have highlighted that mitral valves are enlarged at birth, and continue to enlarge as the heart matures [1,139]. It is therefore hypothesized that mechanical forces applied to the enlarged mitral valve further stimulate connective tissue growth and disorganization, and contribute to valvular enlargement over a lifetime [1]. This was shown effectively by Connell et al., who demonstrated, using an *ex vivo* live tissue bioreactor able to simulate normal MV dynamics, as well as dynamics consistent with MVP, that MVP dynamics caused a decrease in the MV tissue stiffness, an increase in MV thickness, and myxomatous remodeling of the MV [140]. The relation of many MVP-associated mutations and variants to the ciliary gene demonstrated by several groups has also implicated abnormal ciliary mechanosensing by the valve interstitial cell in the pathogenesis of MVP [139,141].

As mentioned above, the stress–strain relationship between the mitral valve and the left ventricle is complex and relies on multiple factors, including the transmitral pressure gradient during ventricular systolic contraction, the geometries of the mitral leaflet and annulus, the degree of leaflet coaptation, and the degree of mitral regurgitation [44]. This is true and of special importance in MVP. *In vitro* LV model studies conducted by Ostli et al. demonstrated that a linear relationship exists between the ventricular pressure and chordae tendineae forces in the native state [9]. Circumstantial clinical evidence exists to support this relationship as the observation that pre-existing hypertension is more prevalent in patients who present with acute idiopathic chordal rupture than in patients who have secondary chordal rupture or patients who present to the hospital with an unrelated condition [142,143]. Annular and leaflet geometries are known to be enlarged in MVP [144,145], yet how these changes in geometry affect subvalvular forces is debated. *In vitro* and *in vivo* models have, to-date, mostly consisted of an isolated flail of a leaflet portion through primary chord severing [146,147]. Attempts to create a model of MVP using mitral leaflet augmentation with pericardial patches to increase the leaflet surface area with no change to the annular size showed that enlargement of the leaflet surface area decreased the secondary chordal forces relative to the native state [9,33,148]. However, these decreases were linked to a change in the relative location of the secondary chordae relative to the coaptation zone: all the models analyzed displayed an increase in leaflet coaptation and secondary chordal insertion sites closer to the coaptation zone—this likely decreased the net forces exerted on the PMs due to a counterbalancing effect on the transduced forces by the opposing leaflet. Other bioreactor studies, including an elegant cross-species Barlow’s model [149], have demonstrated that decreased leaflet coaptation is associated with increased forces on the primary/secondary chordae, as well as the total force exerted on the papillary muscles, and that improved leaflet coaptation significantly decreases these forces [44,149]. The effect of mitral regurgitation (as an additional factor to decreased coaptation) on subvalvular forces is complex. Because MR is associated with a decrease in transmitral pressure [149], it can be inferred that worsening MR will decrease the direct mechanical forces on the chordae tendineae and papillary muscles. However, worsening MR in MVP is usually also associated with enlarged leaflet and annular geometries, decreased leaflet coaptation, or elongated chordae tendineae, all of which can increase the forces experienced by the PMs. For this reason, increased MR is often positively correlated with indirect evidence of increased forces, such as a higher prevalence of left ventricular fibrosis [150,151].

**Figure 12:**
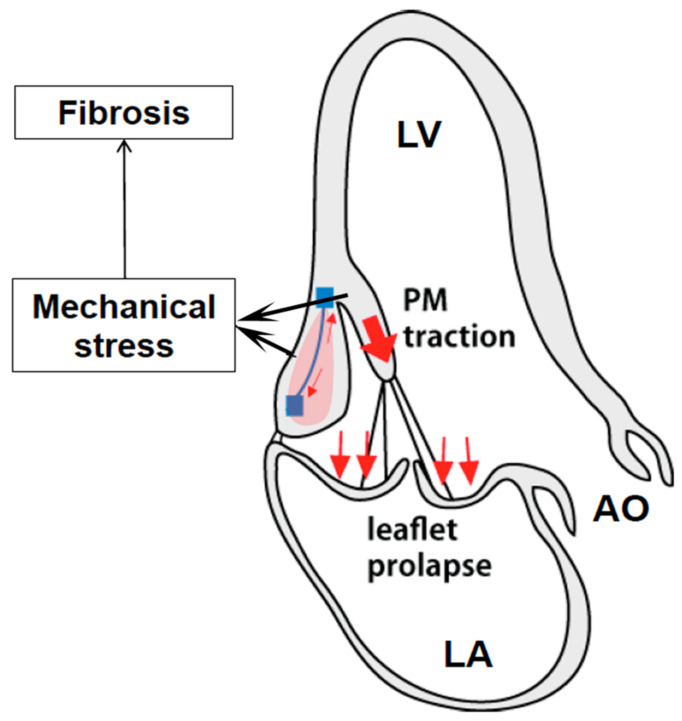
Proposed mechanism of mechanically induced papillary muscle and localized left ventricular fibrosis subsequent of tugging forces exerted by the prolapsing valve. [153].

Historically, secondary complications of MVP were thought to be driven primarily by worsening mitral regurgitation; however, new studies have highlighted that ventricular arrhythmias and sudden cardiac death can occur in MVP patients, even when little to no regurgitation is present [151,152]. It is hypothesized that enlarged mitral valve geometries in MVP and, in particular, decreased leaflet coaptation cause an increase in the applied force to the subvalvular structures tethering the mitral valve to the left ventricle (Figure 12) [153]. It has long been observed that MVP is associated with parallel superior papillary muscle displacement during systole, which is likely indicative of increased mechanical forces applied to the subvalvular apparatus pulling the myocardium superiorly [154]. More recently, MVP has been associated with regionalized left ventricular fibrosis, which localizes to the basal inferolateral myocardial wall and papillary muscle in a pattern suggestive of a mechanical cause resulting from these increases in the tugging forces through the chordae tendineae [151,153].

**Figure 13:**
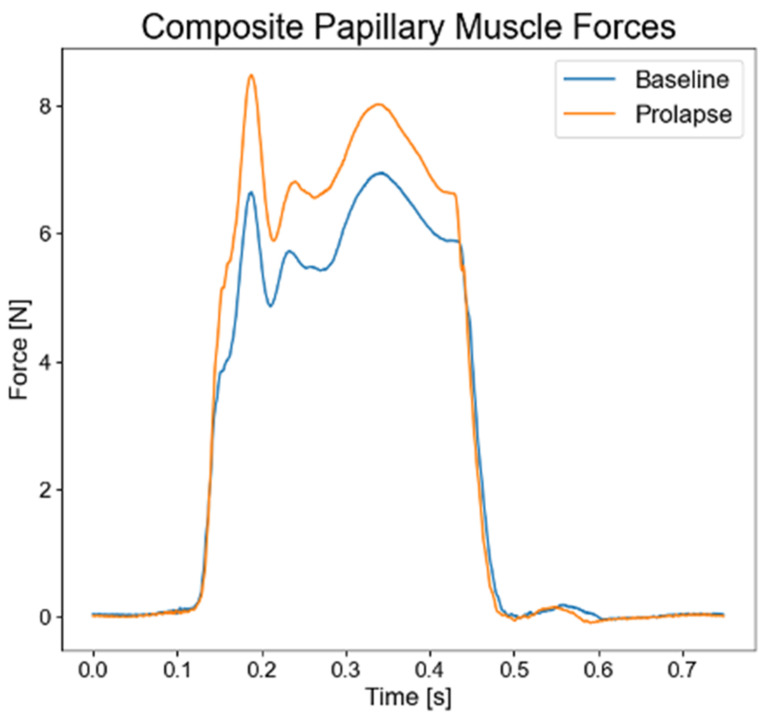
Directly measured papillary muscle forces in physiologic and prolapse conditions [44].

Direct evidence of increased forces on the papillary muscle due to single or bileaflet MVP was demonstrated recently using the elaborate *ex vivo* simulator at the Woo laboratory at Stanford and a novel system to adjust the primary chordae tendineae length without manipulating any other elements of the MV and subvalvular structures (see Figure 13) [44]. Furthermore, utilizing advanced mechanical analysis based on an echocardiography-derived LV strain, it was found that altered longitudinal strain is seen in the basal inferolateral myocardium, situated between the mitral annulus and papillary muscles in patients with MVP compared with healthy controls [155]. In addition, studies have observed a decrease in papillary muscle systolic shortening associated with MVP, which may reflect increases in the tissue stiffness due to either PM fibrosis or opposing stretching forces as a results of the increase in tension from the prolapsing valve [156]. Collectively, it appears likely that the common characteristics of MVP (increased systolic transmitral pressures, larger leaflet/annular areas, decreased leaflet coaptation) lead to an increase in the force applied through the chordae tendineae to the PMs and LV wall, culminating in either chordal rupture or PM/inferobasal fibrosis.

Historically, the primary indication for valve surgery has relied on indications of hemodynamic compromise, such as severe MR, symptoms of heart failure, a new onset of atrial fibrillation, or a decline in echocardiographic contractile function [126]. These guidelines rely heavily on MR as the primary pathology to decide whether and when surgery is indicated; however, the observation that substantial numbers of MVP patients have regionalized fibrosis and lethal ventricular arrhythmias that may occur regardless of MR brings into question whether surgical intervention should also be considered due to other indications of abnormal mechanics of MV-linked structures that may indicate pathologic mechanical forces with potentially catastrophic consequences [150,151,152,153].

### 4.2. Surgical Repair Techniques

**Figure 14:**
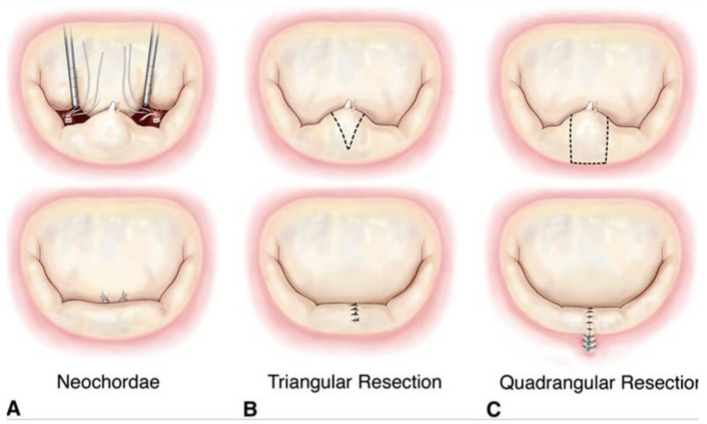
Different leaflet repair techniques for prolapse of the P2 segment. Reprinted with permission from [157]. Copyright 2009, Elsevier.

The optimal surgical repair of MVP would (1) eliminate mitral regurgitation, (2) restore mitral valve and subvalvular geometry, (3) remodel the MV annulus, and (4) ultimately improve leaflet coaptation, all of which would, in turn, normalize the hemodynamics and force distribution to the subvalvular apparatus. In surgical mitral valve repair, the pathologically changed MV leaflets present the first challenge that needs to be tackled. In a comprehensive biomechanical analysis conducted in the Stanford *ex vivo* simulator comparing four different techniques commonly used to surgically eliminate MR (edge-to-edge repair, neochordalplasty, nonresectional remodeling, and leaflet resection technique), it was found that all of these techniques, except edge-to-edge repair, were able to effectively correct mitral regurgitation due to P2 flail [147]. When comparing the techniques that successfully eliminated MR relative to one another, it was observed that the neochord insertion and nonresectional technique caused a decrease in both the primary and secondary chordal forces relative to the resectional technique. The decrease in forces in the neochord technique was hypothesized to result from an increase in the number of chords available for force distribution, while the decrease present in the nonresectional technique was thought to be due to improvements in leaflet coaptation [147]. These studies are consistent with others by the same group, which show that surgical correction of enlarged and redundant leaflet tissue using a nonresectional neochordal technique improves the coaptation area and decreases both the primary and secondary chordal forces, the maximal rate of change of force, and the total papillary muscle force [44,149]. Figure 14 illustrates three common leaflet repair techniques.

Recently, it was shown in an *ex vivo* setting that deviation of only 1 mm from the optimal neochord length results in increases in the papillary muscle forces and residual mitral regurgitation. This was true for elongated and foreshortened neochords, but the effect was more pronounced with elongated neochords [158]. The location of neochord placement during surgical repair can also have different effects on how the forces are balanced in between the valve and ventricle and may have a significant impact on the long-term durability of the repair. *Ex vivo* bioreactor studies investigating the suture anchoring width and leading-edge distance demonstrated that increases in the suturing anchor width and a larger distance to the leading edge both increased the suture pull-out force [159]. Given that increasing the leading-edge distance too much would result in a decreased leaflet coaptation length, Pandya and colleagues ultimately found that, from a biomechanical point of view, insertion of artificial chords at 6 mm from the leading edge and with a width of 10 mm would be the optimal configuration. However, all the suture pull-out forces observed in their experiments vastly exceeded the forces usually observed for the primary or secondary chordae tendineae [159]. A recent study by Fernández and colleagues showed that different techniques of anchoring the neochords at the papillary muscles may influence neochordal biomechanics; however, this is not the case when reviewing the loading conditions within the physiological range [160]. Classically, neochords are affixed to the papillary muscle tips, restoring more normal mechanics; however, recent biomechanical evidence suggests that alternative implantation into the apical or posterior myocardium may provide satisfactory mitral valve repair, as well [38,146,161]. *In vivo* implementation of the force measurement in the clinical trans-apical implantation of novel apically fixated artificial chords demonstrated that the force on one artificial chord connected to the P2-segment of the MV decreases as the artificial chords are implanted, coinciding with the achievement of optimal leaflet coaptation and minimization of MR [161]. However, no comparative studies regarding the location of neochord placement were performed in that setting. In *ex vivo* bioreactor studies, apical implantation was associated with increased forces on the chordae and on the neochords, as well as a higher maximum rate of change of force when compared with implantation in the PM tips [146,162]. A notable increased rate of reoperations after transapical neochord repair when compared to the standard surgical technique may suggest that these higher forces and different force profiles relate to earlier neochord failure [146,162,163,164]. *In vivo* studies conducted in pigs that compared papillary vs. apical fixation of artificial chords did not find any differences in the neochordal tension amount, but did find differences in the maximal rate of change of tension between the two groups [162]. More recently, anchoring of neochords in the posterior LV myocardium to correct MVP has been found to restore primary and secondary chordal forces to baseline levels in the Stanford *ex vivo* simulator; however, this technique was not compared with papillary or apical fixation [38]. Posterior anchoring may be of special interest for the future development of devices that can be used without cardioplegic arrest in more frail patients. Of note, methods to lower forces that result from apical implantation are being developed currently in *ex vivo* bioreactors and may support more widespread use of this off-pump technique in patients not eligible for standard repair surgery [165].

In the standard surgical approach, in addition to the leaflet repair techniques, mandatory concomitant annuloplasty is used to reinforce healthy annular dimensions and durably improve leaflet coaptation [166]. Multiple formats of annuloplasty rings exist, including rigid, semi-rigid, and flexible types, as well as partial or complete ring or band formats, and flat or saddle-shaped geometries [166]. While rigid, semi-rigid, and flexible annuloplasty rings have been shown to improve mitral annular circumference and leaflet coaptation compared to no annuloplasty ring, only flexible annuloplasty rings were found to decrease both the chordal forces and maximum rate of change of forces compared to pre-procedural conditions [65,167]. In contrast, rigid annuloplasty rings were associated with a higher rate of change of force compared with pre-procedural conditions, which may reflect the rigidity of the implant [167]. Saddle-shaped annuloplasty rings were shown to improve the distribution of forces across the annulus when compared with flat annuloplasty rings, indicating that reinforcement of the annulus in a more physiologic state improves how the forces are handled by the mitral valve [66]. Finally, while there are no differences in repair durability, flexible partial (posterior) annuloplasty bands as opposed to complete semi-rigid rings may be advantageous with regard to more physiologic annular dynamics (diastolic anterior expansion), less energy loss within the ventricle and the aortic outflow tract, and lower MV pressure gradients, especially in annuli <30 mm [166,168,169,170,171].

Transcatheter edge-to-edge repair (TEER), already described above for its use in IMR, was originally developed for MVP-therapy and is based on surgical edge-to-edge repair, as described by Alfieri and colleagues [172]. As shown by Paulsen et al., of all the available surgical leaflet repair methods, this technique has the least favorable hemodynamic and biomechanical results in MVP repair [147]. However, the simplicity of this approach has driven the development of the original MitraClip and subsequent device generations, initially thought to be used in patients not eligible for surgery. As efforts increase to drive more widespread application even in low-risk surgical candidates, the biomechanical effects of TEER are currently a topic of high interest to the community. As elucidated above, one of its main limitations is the fact that only the leaflets, but no other components of the MV–LV apparatus are targeted. Bhattacharya and He conducted a biomechanical analysis of annular tension after TEER in posterior and anterior leaflet prolapse in an *ex vivo* bioreactor and found that TEER does not restore annular tension to physiologic values, and therefore may leave patients at increased risk of progressive annular dilation and recurrent MR [173]. Using the Stanford *ex vivo* simulator [7,32,42,106,147,149,167], it was recently shown that, in contrast to surgical neochordal repair, application of a MitraClip does not normalize the forces exerted on the PMs, despite similar reductions in MR [174]. Computationally, Zhang and colleagues [2019] also demonstrated that this device induces abnormal strains in the ventricular wall. Taking into account the current evidence for abnormal mechanics in MVP due to increased traction forces that are independently associated with the presence of localized fibrosis and the occurrence of life-threatening ventricular arrhythmias [155], this may have larger implications for long-term outcomes; however, it remains a topic of much needed ongoing research efforts.

## 5. Knowledge Gaps

In light of the evidence provided above, it is clear that biomechanical engineering approaches correlated with biological studies in a variety of models have the capacity to address multiple research opportunities in mitral valve disease that will improve medical and surgical therapies [175]. The following questions require more definitive answers and are therefore potential focus areas of future research in this field: (1) What is the biomechanical impact of valve-focused versus ventricular-focused (subvalvular) repairs for IMR? (2) What are the potential approaches to measure and modulate MV thickness and stiffening to prevent or reduce IMR? (3) Which aspects of the MV–LV apparatus can and should be treated via transcatheter approaches to provide biomechanical improvements and durable results? (4) What is the role of abnormal mechano-sensing in the pathogenesis of degenerative MV disease? (5) What drives LV fibrosis and ventricular arrhythmias in patients with MVP? (6) Can transcatheter approaches truly accomplish biomechanical normalization of the MV–LV relationship in MVP?

## 6. Conclusions

This review article has described in detail how bioengineering approaches continue to provide insights into MV–LV biomechanics in healthy and diseased conditions, and how they can be improved. For decades, ever improving, high-throughput testing has been utilized through complementary *ex vivo*, computational, and *in vivo* studies, allowing examination of the interaction of the MV, LV, and annular structures in a controlled manner with biomechanical techniques. Further definition of the above-mentioned aspects of the MV–LV relationship and identification of the tissue properties affecting cardiac fluid dynamics can provide novel therapeutic targets through the translational potential of biological–mechanical studies. In conclusion, the development of optimal treatment strategies will always require bioengineering concepts and modelling conditions that can test whether physiologic valve and ventricular function and their interactions are indeed restored sufficiently.

## Data Availability

Not applicable.
